# Supplementary material for: Haplosaurus computes protein haplotypes for use in precision drug design
Source: Nat Commun. 2018 Oct 8;9:4128. doi: 10.1038/s41467-018-06542-1 (PMC6175845; doi:10.1038/s41467-018-06542-1)
Supplement: Supplementary file 1 — Supplementary Information [file 41467_2018_6542_MOESM1_ESM.pdf]

SUPPLEMENTARY INFORMATION

Haplosaurus Computes Protein Haplotypes for use in Precision Drug Design.

Spooner et al.

SUPPLEMENTARY FIGURES

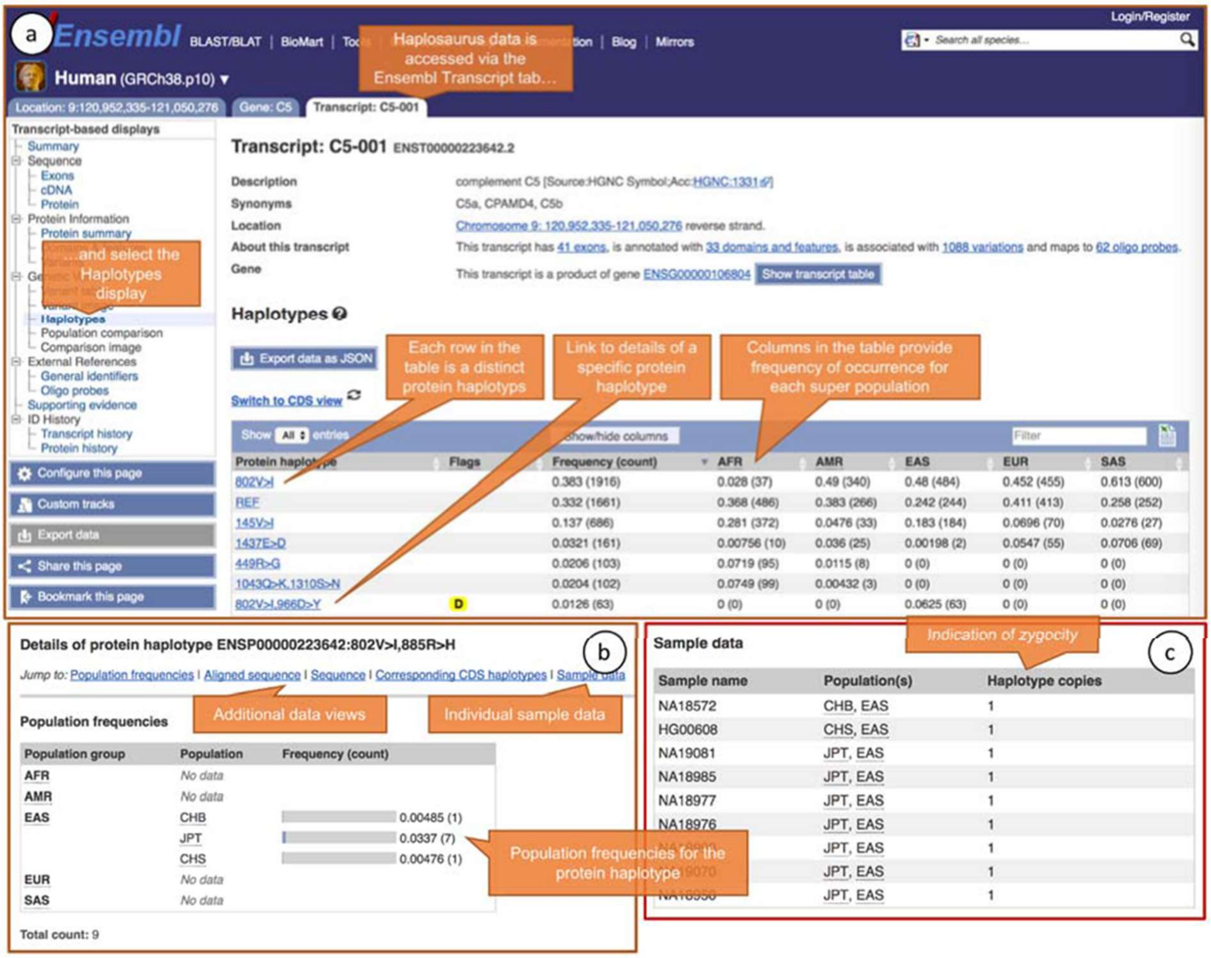

Supplementary Figure 1. Ensembl website's Transcript Haplotype view for gene *C5*. http://grch37.ensembl.org/Homo_sapiens/Transcript/Haplotypes?t=C5-001. a) shows how the view is accessed for the gene transcript via the left-hand navigation panel, and the summary table of all protein haplotypes for the gene transcript. b) shows the population group and population frequencies for a selected protein haplotype. c) shows details at the sample level for a selected protein haplotype.

a

b

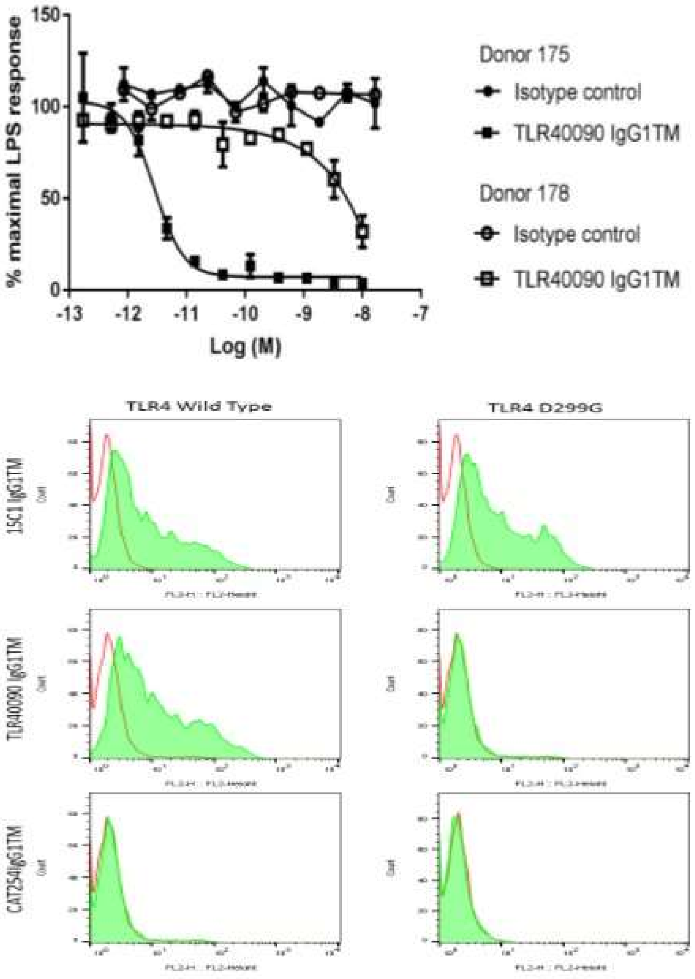

Supplementary Figure 2. Biological activity and binding of mAbs for TLR4.
a) Human TLR4 antibody (TLR40090) inhibition of LPS induced TNFα release in PBMCs of donors heterozygous for haplotype TLR4:299D>G, 399T>I compared to donors expressing reference sequence TLR4, b) binding to cell lines transiently transfected with reference sequence or TLR4:299D>G. a) PBMC were incubated with LPS derived from S. Minnesota for 1ng/ml after pre-incubation for 30 mins with antibodies at concentrations indicated. TNFα was measured from supernatant by ELISA and expressed as % maximal signal for LPS alone. Here we show an example experiment where donors were tested in parallel in the same experiment. The effect of antibodies was tested in Donor 175 n=7 and Donor 178 n=6 times. Donor 175 was typical of 14 responder donors tested, Donor 178 was typical of 3 low-responder donors tested. b) HEK293 cells were transiently transfected with either Reference sequence TLR4 or TLR4:299D>G and binding of TLR40090 was measured by flow cytometry. Control antibody CAT254 IgG1TM did not bind any cells; and control anti-TLR4 antibody 15C1 bound to both cell lines confirming expression. TLR4:299D>G abolished binding to TLR40090. Direct impact of TLR4:299D>G was later confirmed with the crystal structure of the human TLR4 (D299G/T399I). MD-2.LPS complex shows local structural differences around D299G but not around the T399I SNP site. The differences lead to conformational changes affecting the binding site of the ligands further away[1].

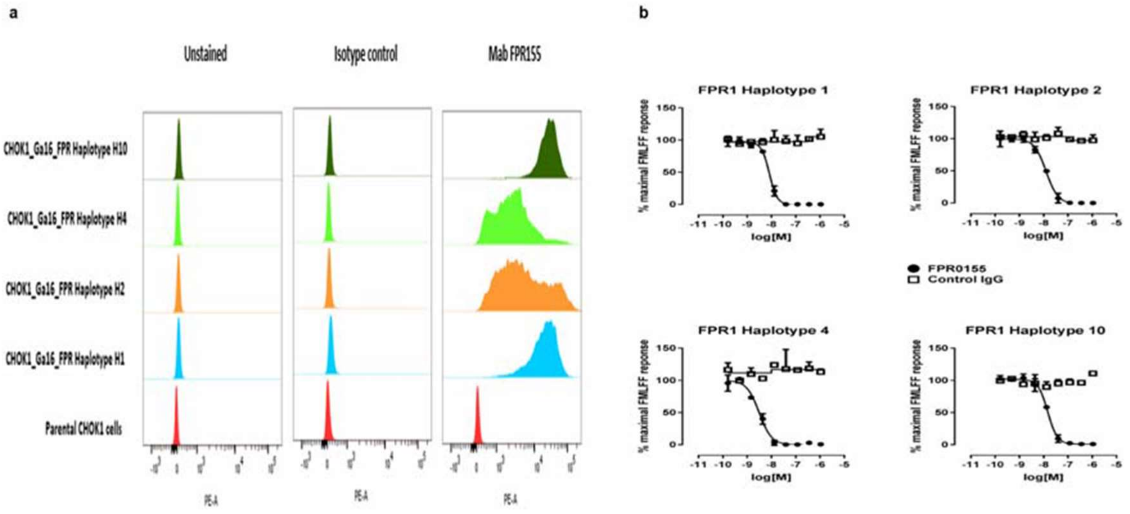

Supplementary Figure 3. Biological activity and binding of mAbs for FPR1.
a) Flow cytometric analysis of MAb FPR155 binding, compared to isotype control and unstained control, to four FPR1 haplotype variant cell lines compared to untransfected CHO parental cell line. Fpro0155 binds to all four FPR haplotypes H1, H2, H4 and H10 covering 98% of the possible extracellular variations found in all listed FPR haplotypes (see Figure 6 main text). FACS staining was carried out using Fpro0155 IgG1 and control IgG1 with parental, and overexpressing FPR Haplotype H1, H2, H4 and H10 CHOK1 cell lines (coexpressing the promiscuous G protein Galpha16). b) Complete inhibition by MAb FPR0155 of formyl peptide-induced responses in 4 variant FPR1 reporter cell lines Complete inhibition by MAb FPR0155 antibody, compared to isotype control, of 400pM FMLFF-induced calcium signalling was seen in all four haplotype variant cell lines of CHOK1-Gα16 FPR1 reporter cells. Data shown are from one experiment performed in triplicate, typical of 2 independent experiments. Potency cannot be directly compared due to differences in absolute receptor expression levels in the different cell lines.

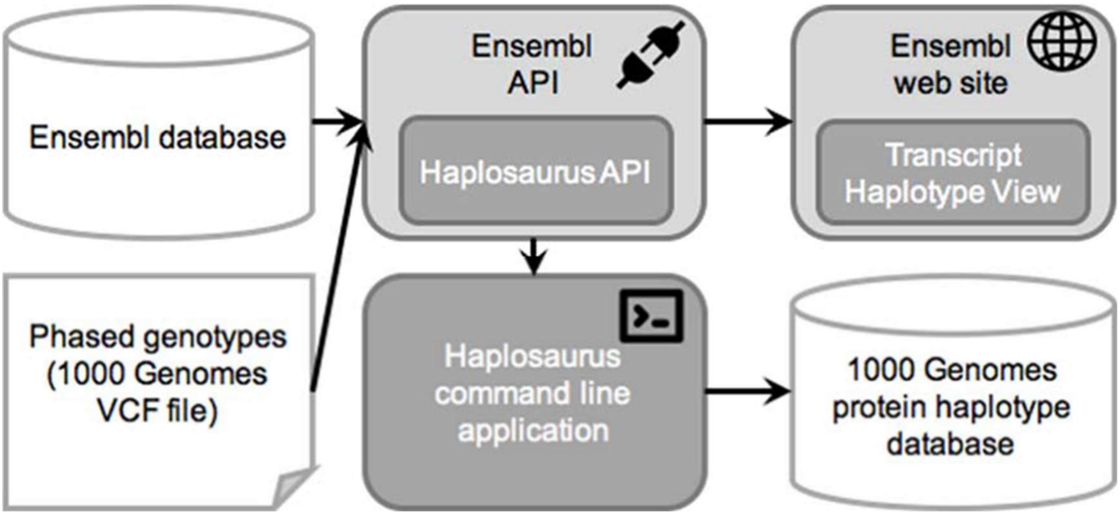

Supplementary Figure 4. Haplosaurus bioinformatics software. Architecture of Haplosaurus and associated bioinformatics software (grey rectangles) for inferring protein haplotypes from phased genotypes. White shapes indicate data inputs/outputs.

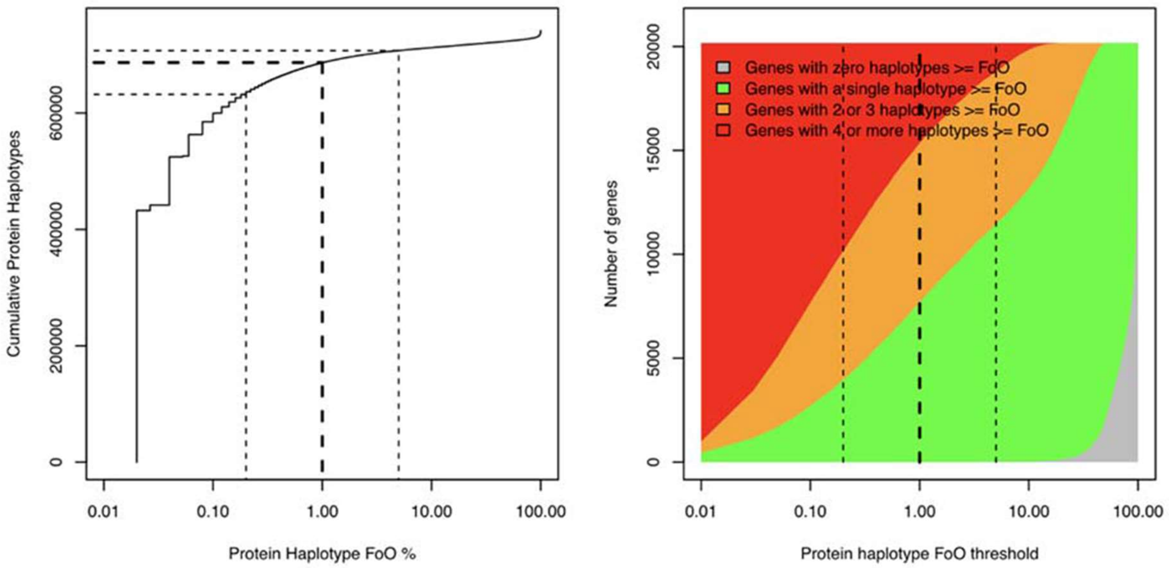

Supplementary Figure 5. Distributions of protein haplotype by Frequency of Occurrence (FoO). On the left is a cumulative plot of FoO for each of the 741,639 protein haplotypes of the 20,166 canonical protein coding transcripts. On the right is the number of genes in each category of protein-haplotypes-per-gene (zero, one, two or three and four or more) above FoO threshold (x-axis). For both plots the thick dashed line represents the FoO 1% significance threshold, thin dashed lines represent alternative FoO thresholds at 0.2% and 5%.

SUPPLEMENTARY TABLES

Test Type	Test Description
SUBSTITUTION	Single base substitution of each of the three possible alternative bases at each position
1BP INS	Inserts a base after each position cycling through ACGT as the inserted base
1BP DEL	Deletes the second of every pair of bases for each position
2BP INS	Inserts two bases after each position, the first cycling through ACGT for each inserted base and the second being a random base from ACGT
2BP DEL	Deletes the second and third of every three bases for each position
STOP	Inserts a stop codon 5 bases after the start of the sequence (Note that the VCF format for this test was known to be incorrect)
COMPLEX SUBS	Substitute two positions 10 bases apart with non-reference random bases, repeating every 10 bases
COMPLEX INDEL	For two pairs of bases 10 bases part, delete the second base of both pairs, repeating every 10 bases

Supplementary Table 1. Description of types of test cases listed in the test log files. Each test type is applied independently for every possible position along the original test DNA sequence.

Gene description	Total	Identical	Different	Comments
IGHJ6, single exon, minus strand	436	433	2 (#244, #365)	Test #425 skipped by Haplosaurus due to invalid VCF format
IGKV4-1, two exons, plus strand	5447	3867	4 (#2495, #3028, #4008, #4541)	1576 tests skipped by Haplosaurus as intronic

Supplementary Table 2. Summary of test outcomes for the 5883 test cases run across two genes. The test numbers shown prefixed with # correspond to those listed in the test log files. Cases listed as different were due to known differences in the treatment of splice sites by the two methods.

SUPPLEMENTARY REFERENCES

1. Ohto, U., Yamakawa, N., Akashi-Takamura, S., Miyake, K. & Shimizu, T. Structural analyses of human Toll-like receptor 4 polymorphisms D299G and T399I. The Journal of biological chemistry 287, 40611-40617 (2012).
