# Supplementary material for: Haplosaurus computes protein haplotypes for use in precision drug design
Source: Nat Commun. 2018 Oct 8;9:4128. doi: 10.1038/s41467-018-06542-1 (PMC6175845; doi:10.1038/s41467-018-06542-1)
Supplement: Supplementary file 3 — Description of Additional Supplementary Files [file 41467_2018_6542_MOESM3_ESM.pdf]

Description of Additional Supplementary Files

File Name: Supplementary Data 1
Description: Protein haplotypes by gene.

File Name: Supplementary Data 2
Description: Protein haplotypes distributions. Protein haplotypes by gene classified by 1000genome super populations; per selected druggable gene class and per gene targets to biotherapeutics according to their clinical trial phase.

File Name: Supplementary Data 3
Description: Protein haplotypes by gene classified by DGIdb category.

File Name: Supplementary Data 4
Description: Data used to test/validate Haplosaurus.

File Name: Supplementary Data 5
Description: Fasta protein sequences of all protein haplotypes in 1000 Genomes.
